# Antifungal activity of liriodenine on agents of systemic mycoses, with emphasis on the genus *Paracoccidioides*

**DOI:** 10.1590/1678-9199-JVATITD-2020-0023

**Published:** 2020-10-28

**Authors:** Adriele Dandara Levorato Vinche, Iván de- la-Cruz-Chacón, Alma Rosa González-Esquinca, Julhiany de Fátima da Silva, Gisela Ferreira, Daniela Carvalho dos Santos, Hans Garcia Garces, Daniela Vanessa Moris de Oliveira, Camila Marçon, Ricardo de Souza Cavalcante, Rinaldo Poncio Mendes

**Affiliations:** 1 Department of Tropical Disease and Imaging Diagnosis, Botucatu Medical School, São Paulo State University (UNESP), Botucatu, SP, Brazil.; 2 Universidad de Ciencias y Artes de Chiapas, Tuxtla Gutierrez, Chiapas, Mexico.; 3 Institute of Biosciences of Botucatu, São Paulo State University (UNESP), Botucatu, SP, Brazil.; 4 Faculdade de Ciências da Saúde, Universidade do Oeste Paulista (Unoeste), Presidente Prudente, SP, Brazil.

**Keywords:** Liriodenine, Paracoccidioidomycosis, Systemic mycosis, Antifungal compounds, Antimicrobial compounds, Medicinal plants

## Abstract

**Background:**

Endemic systemic mycoses remain a health challenge, since these opportunistic diseases are increasingly infecting immunosuppressed patients. The simultaneous use of antifungal compounds and other drugs to treat infectious or non-infectious diseases has led to several interactions and undesirable effects. Thus, new antifungal compounds should be investigated. The present study aimed to evaluate the activity of liriodenine extracted from *Annona macroprophyllata* on agents of systemic mycoses, with emphasis on the genus *Paracoccidioides*.

**Methods:**

The minimum inhibitory concentration (MIC) and minimum fungicide concentration (MFC) were determined by the microdilution method. The cellular alterations caused by liriodenine on a standard *P. brasiliensis* (Pb18) strain were evaluated by transmission and scanning electron microscopy.

**Results:**

Liriodenine was effective only in 3 of the 8 strains of the genus *Paracoccidioides* and in the *Histoplasma capsulatum* strain, in a very low concentration (MIC of 1.95 μg.mL^-1^); on yeasts of *Candida* spp. (MIC of 125 to 250 μg.mL-1), including *C. krusei* (250 μg.mL^-1^), which has intrinsic resistance to fluconazole; and in *Cryptococcus neoformans* and *Cryptococcus gattii* (MIC of 62.5 μg.mL^-1^). However, liriodenine was not effective against *Aspergillus fumigatus* at the studied concentrations. Liriodenine exhibited fungicidal activity against all standard strains and clinical isolates that showed to be susceptible by *in vitro* tests. Electron microscopy revealed cytoplasmic alterations and damage to the cell wall of *P. brasiliensis* (Pb18).

**Conclusion:**

Our results indicate that liriodenine is a promising fungicidal compound that should undergo further investigation with some chemical modifications.

## Background

Endemic systemic mycoses remain a health challenge, especially because these opportunistic affections are continuously infecting immunosuppressed patients. The simultaneous use of antifungal compounds and other drugs to treat infectious or non-infectious diseases has led to several interactions and undesirable effects. Therefore, alternative antifungal compounds should be found. The present study aimed to evaluate the activity of liriodenine extracted from *Annona macroprophyllata* on agents of systemic mycoses, particularly the genus *Paracoccidioides*.

Paracoccidioidomycosis (PCM) is a systemic mycosis limited to Latin America and is caused by different species of the genus *Paracoccidioides* - *P. brasiliensis* complex, *P. americana*, *P. restrepiensis*, *P. venezuelensis,* and *P. lutzii* [1-5]. These fungi are the main causative agents of endemic mycoses in this region [1]. PCM affects mainly rural workers, leads to severe pulmonary sequelae in most cases, is considered a neglected disease, and is the 8^th^ cause of death among the chronic diseases in Brazil [6-10].

The etiologic agents of other systemic mycoses were also studied due to their prevalence and importance, namely:

- histoplasmosis, an endemic disease with the highest incidence in AIDS patients [11];
- candidiasis, for its high incidence in neutropenic patients and as the main cause of nosocomial infections [12];
- cryptococcosis, because of the high incidence in AIDS patients and the difficulties in its treatment [13,14];
- aspergillosis, for its incidence in tuberculosis patients with pulmonary cavities, and in neutropenic patients developing invasive disease [15].

The incidence of fungal infections has increased dramatically in recent decades, posing serious problems for the management of immunosuppressed patients with systemic mycoses [16-19]. During the same period, a wide gap has occurred in the development of antimicrobial compounds: few antifungal and many antibacterial compounds [20,21].

**Figure 1.**
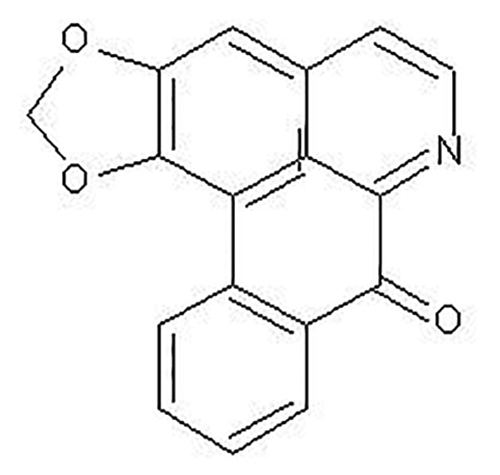
Structure of liriodenine.

The development of new drugs derived from plant extracts have increased in the last few years [22-24]. Plants produce different compounds with biological properties and activity against diseases of different etiologies, such as inflammatory diseases, infectious diseases, and cancer. Liriodenine (Fig. 1) is an oxoaporphine alkaloid, yellow needle, fluorescent, plain, with an oxo group in position 7. Liriodenine has antibacterial, antifungal, antiprotozoal and cytotoxic properties, is isolated from plants of several families, including the *Annonaceae* [25-31]. Its antifungal activity was observed on *Candida albicans* (ATCC 10231), *Aspergillus niger* (ATCC 16888), *Trichophyton mentagrophytes* (ATCC 9972), and the phytopathogenic fungi *Aspergillus glaucus* and *Rhizopus stolonifer* [25,26,28,30].

The objective of the present study was to evaluate the antimicrobial activity of liriodenine against fungi of the genus *Paracoccidioides* and other causative agents of systemic mycoses *in vitro*, as well as observe the morphological alterations caused by this compound in a standard *P*. *brasiliensis* (Pb18) strain. 

## Materials and Methods

### Ethics statement

This study was approved by the Research Ethics Committee of Botucatu Medical School (FMB) of São Paulo State University (UNESP), Botucatu, SP, Brazil, under protocol number 2.257.787. 

### Liriodenine

Liriodenine was obtained from the Laboratory of Plant Physiology and Chemistry, Institute of Biological Sciences, Chiapas University of Arts and Sciences. Two of the authors, ICC and ARGE, are researchers from the Service of Plant Physiology and Chemistry and were responsible for supplying the sample. Liriodenine was extracted from *Annona macroprophyllata*, which is planted for commercial purposes. Moreover, the present research did not involve any endangered or threatened species.

*Plant materials*

 Root bark (300 g) of *Annona macroprophyllata* (≡ *Annona diversifolia*) located at the geographical coordinates 16º46’34” N 93º07’14” W were collected in September 2011 in Tuxtla Gutiérrez, Chiapas, Mexico. The taxonomic sample was under reference voucher #352 of the Eizi Matuda Herbarium (HEM) of the Chiapas University of Arts and Sciences, Mexico. 

*Liriodenine extraction*

Root barks were dried at room temperature (at 25-30 ºC for one week). After thorough grinding, the plant material was moistened with a saturated sodium carbonate (Na_2_CO_3_) solution and left to dry for 48 hours at room temperature. Alkaloids were extracted with chloroform (CHCl_3_) by constant stirring for 1 hour and then filtered and washed with distilled water. The CHCl_3_ phases were extracted into a 1 M hydrochloric acid (HCl) solution before being alkalinized to pH 9.5 with a saturated solution of Na_2_CO_3_. The alkaline solution was then reextracted with CHCl_3_, dried with anhydrous sodium sulfate (Na_2_SO_4_), filtered and evaporated at approximately 25 °C to obtain total alkaloids.

From 300 g of dried and powdered roots, 1200 mg of total alkaloids were extracted and a solid yellow precipitate was obtained. This was purified by fractionated crystallizations with a CHCl_3_-MeOH system to obtain 310 mg of solid yellow needles, which indicated positive results in both the Dragendorff’s and the Mayer’s tests.

Liriodenine standard was obtained as yellow needles from CHCl_3_, with a melting point (m.p.) of 280-282 °C. The product was identified as liriodenine by Dr. Miguel Angel Pérez Farrera from the herbarium of the National Autonomous University of Mexico. Spectrometric identification data were reported in De La-Cruz-Chacón and González-Esquinca [29].

*Chemicals*

Hydrochloric acid, methanol, chloroform, water, sodium carbonate and anhydrous sodium sulfate were purchased from J.T. Baker, USA. The methanol and water used were HPLC grade.

### Microorganisms

Strains and clinical isolates of agents of systemic mycoses were evaluated in this study: 8 from the genus *Paracoccidioides*, 5 of the genus *Candida*, 2 of the genus *Cryptococcus* - 1 of the *Cryptococcus neoformans* complex and 1 of the *Cryptococcus gattii* complex, 1 of *Histoplasma capsulatum*, and 1 *Aspergillus fumigatus*.

Strains obtained from the American Type Culture Collection (ATCC) and clinical isolates of the Laboratory of Research on Tropical Diseases, Botucatu Medical School (FMB), São Paulo State University (UNESP), SP, Brazil, were used in this study and include the following fungi of the genus *Paracoccidioides*: *P. brasiliensis* - Pb18 (fungal collection of FMB/UNESP; isolated from a PCM patient with the chronic form); Pb192 (fungal collection of FMB/UNESP; unknown clinical form); Pb339 (ATCC 3206; chronic clinical form); *P. lutzii* - PL01 (ATCC MYA-826; unknown clinical form); and PL8334 (unknown clinical form). Isolates number 234, 326 and 531 were obtained from PCM patients admitted in the Infectious Diseases Ward of Botucatu Medical School, UNESP, cultivated in the Research Laboratory on Tropical Diseases and identified at the Research Laboratory of Microbiology, Botucatu Biosciences Institute, UNESP. Isolate number 234 was obtained from the sputum of a PCM patient with the chronic form (August 8^th^, 2009), isolate number 326 from the oral mucosa of a PCM patient with the acute/subacute form (February 8^th^, 2012), and isolate number 531 from the scrotum of a PCM patient with the chronic form (January 9^th^, 2017). The following strains were used as representatives of fungi that can cause other systemic mycoses: *Candida albicans* (ATCC 90028), *Candida tropicalis* (ATCC 750), *Candida glabrata* (ATCC 90030), *Candida parapsilosis* (ATCC 22019), *Candida krusei* (ATCC 6258), *Cryptococcus neoformans* (ATCC 28957), *Cryptococcus gattii* (AFLP4), *Histoplasma capsulatum* (ATCC 26029), and *Aspergillus fumigatus* (ATCC 7100).

### Molecular characterization and phylogenetic analysis of clinical isolates of fungi of the genus Paracoccidioides

The clinical isolates from PCM patients were identified by sequencing the exon 2 region of gp43 glycoprotein, a highly variable genetic region which allows species differentiation [3]. DNA extraction was performed following the "Quick CTAB" protocol [32], and exon 2 of gp43 was amplified by PCR in a Veriti Thermocycler (Applied Biosystems, Foster City, CA, USA) using primers gp43-E2F (CCAGGAGGCGTGCAGGTGTCCC-3') and gp43-E2R (GCCCCCTCCGTCTTCCATGTCC-3') [33]. To perform the amplification, GoTaq® Green Master Mix (Promega, Madison, WI, USA) was used with a 25 μL reaction volume containing 3 μL of genomic DNA (300 ng/μL) and 0.5 μM of each primer. Cycling was performed as follows: initial DNA denaturation at 94 °C for 5 minutes; 35 cycles of denaturation at 94 °C for 1 minute, annealing at 58 °C for 1 minute, extension at 72 °C for 1 minute and a final extension at 72 °C for 10 minutes.

After confirming the amplification by visualization on a 1.5% agarose gel, the PCR product was purified using ExoProStar (GE Healthcare, Milwaukee, WI, USA) and sequenced on an ABI 3500 DNA analyzer (Applied Biosystems, Foster City, CA, USA) according to the manufacturer's instructions. The quality of the sequences was verified by sequencing analysis software (Applied Biosystems), and only nucleotides with Phred ≥ 20 were included. Sequencing editing and phylogenetic analysis were performed using MEGA v7.0 software [34]. Molecular identification was performed by the Basic Local Alignment Search Tool (BLAST) of the National Biotechnology Information Center (NCBI). Sequence alignments were performed using the Clustal W algorithm. Phylogenetic construction was performed by the maximum likelihood (ML) method [35] with 1000 bootstrap replications and using *P. lutzii* as an external group.

The sequences reported in this paper have been deposited in the GenBank database under the following accession numbers: MH367527, MH 367528, MH367529 and MH367531.

### Susceptibility tests

Susceptibility testing was performed by determining the minimum inhibitory concentration (MIC) by the microdilution method. The MIC of liriodenine was determined for all microorganisms and compared to that of standard antifungal agents (fluconazole - FLC and amphotericin B - AmB) for each genus. RPMI 1640 medium (Sigma) was used without compounds or solvents as a control for growth and sterility. Dimethyl sulfoxide (DMSO) solvent was used as a control for toxicity at the same volume used in the assay. The microdilution test for fungi of the genus *Paracoccidioides* was performed using the protocol proposed by de Paula and Silva et al. [36]. For yeasts, the microdilution method was used according to the specifications of the European Committee on Antimicrobial Susceptibility Testing (EUCAST) [37]. Susceptibility testing of filamentous fungi was performed according to document M38-A2 of the Clinical and Laboratory Standards Institute (CLSI, 2008) [38]. The susceptibility of *Histoplasma capsulatum* was evaluated by the microdilution method according to document M27-A2 of the CLSI (2002), modified by Brilhante et al. [39].

Liriodenine was dissolved in dimethyl sulfoxide. Serial dilutions were prepared in RPMI 1640 medium, maintaining a constant volume of 1 mL per tube. Liriodenine was tested at 10 concentrations ranging from 500 to 0.97 μg.mL^-1^. Volumes of 100 μL of each dilution were distributed in 96-well microplates. The commercial antifungals amphotericin B (Sigma) and fluconazole (Sigma) were included as controls. The final concentrations ranged from 16 to 0.03 μg.mL^-1^ for amphotericin B and from 128 to 0.25 μg.mL^-1^ for fluconazole. The suspensions of yeast-phase cells of the genus *Paracoccidioides* were adjusted to a final concentration of 0.5x10^3^ to 2.5 x 10^3^ cells/mL in RPMI 1640 medium supplemented with 2% glucose. The plates were incubated for 72 hours at 37 ºC under shaking at 150 rpm. After 48 hours of incubation, 20 μL Alamar Blue dye (Invitrogen) was added to all wells, and the plates were incubated for an additional 24 hours. The tests were carried out in triplicate for all fungi. The MIC was determined by the observation of staining after 72 hours of incubation. The cutoff point was the lowest concentration of liriodenine that caused a significant reduction (≥ 50% inhibition) in growth when compared to the control. For fluconazole, the MIC endpoint was defined as the lowest drug concentration resulting in at least 50% growth reduction compared with the control, while that of amphotericin B was the lowest concentration that resulted in at least 90% qrowth reduction compared with the positive control. The plates were read from the highest to the lowest concentration.

### Minimum fungicide concentration (MFC)

After the determination of MICs, subcultures were prepared from all microplate wells containing the concentrations of liriodenine ≥ to that of the MICs. For fungi of the genus *Paracoccidioides*, aliquots (100 µL) of each well were removed and plated on brain heart infusion broth (Difco) containing 0.5% gentamicin, 4% horse serum, and culture filtrate of strain Pb339. Sabouraud agar (Difco) was used for the other fungi. The plates were incubated according to the specific time of each microorganism. The MFC was defined as the lowest concentration of liriodenine resulting in ≤ 1 visible colony on the culture medium after the incubation period.

### Transmission electron microscopy

Transmission electron microscopy (TEM) was performed at the Center of Electron Microscopy of São Paulo State University (UNESP), Botucatu Biosciences Institute, Botucatu, SP, Brazil.

Strain Pb18 was treated with 31.2 μg.mL^-1^ of liriodenine in a 24-well plate. The final volumes of liriodenine and inoculum were adjusted to 1 mL, and Karnovsky’s fixative was added after 48 hours of incubation at 37 ºC. After this period, the material was removed from the fixative and washed three times for 5 minutes each in 0.1 M phosphate buffer, pH 7.3. The material was immersed in osmium tetroxide 0.1 M, pH 7.3, for 2 hours. Next, the material was washed three times for 10 minutes each in distilled water and immersed in 0.5% uranyl acetate for approximately 2 hours. After dehydration in an increasing acetone series, a mixture of Araldite® resin + 100% acetone (1:1) was added, and the material was left to stand for 12 hours at room temperature. Pure resin was added for approximately one hour at 37 ºC, and the material was embedded. Ultrathin (90 nm) sections were cut from the blocks and counterstained with uranyl acetate in 50% alcohol for 20 minutes, followed by counterstaining with lead citrate for 10 minutes. The sections were observed under a Tecnai Spirit transmission electron microscope (FEI Company).

### Scanning electron microscopy

 For scanning electron microscopy (SEM) performed at the same center, strain Pb18 was cultured in RPMI 1640 medium with 2% glucose and treated with the MIC of liriodenine on coverslips coated with poly-L-lysin for 48 hours at 37 ºC. After this period, the medium was discarded, and the material was fixed with 2.5% glutaraldehyde in 0.1 M phosphate buffer, pH 7.3, for 30 minutes. The fixative was removed from the wells, and the coverslips were washed three times for 5 minutes each in distilled water. The coverslips were immersed in 0.5% osmium tetroxide 0.1 M, pH 7.3, in distilled water for 40 minutes. The osmium tetroxide was then discarded, and the coverslips were washed again three times for 10 minutes in distilled water. After dehydration in an increasing alcohol series, the coverslips were critical-point dried, mounted on stubs, and sputter coated. The images were obtained with a Zeiss EVO LS 15 microscope.

## Results

### Phylogenetic analysis of clinical isolates

**Figure 2.**
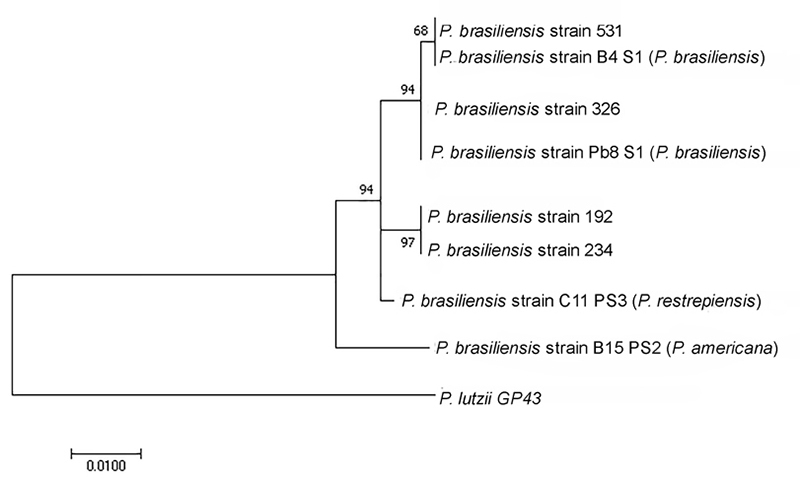
Molecular phylogenetic analysis by the maximum likelihood method using exon 2 of the gp 43 region**.** The evolutionary history was inferred using the maximum likelihood method based on the Kimura 2-parameter model. The tree with the highest log likelihood is shown. The percentage of trees in which the associated taxa clustered together is shown next to the branches. The tree is drawn to scale, with branch lengths measured in the number of substitutions per site. The analysis involved 11 nucleotide sequences. Evolutionary analyses were conducted in MEGA7. The species names were concordant with the latest accepted taxonomies for the species (old and recent species names).

Figure 2 shows the phylogenetic analysis of the exon 2 region of gp43. Clinical isolate 326 was grouped together with strains deposited as *P. brasiliensis* S1a, isolate 531 was grouped together with strains deposited as *P. brasiliensis* S1b, and isolate 234 was grouped together with strains deposited as PS3 (presently called *P. restrepiensis*). Strain 192, also used as a standard strain, was grouped with PS3.

### Minimum inhibitory concentration

Table 1 shows the results of the antifungal activity of liriodenine isolated from *Annona macroprophyllata*.

**Table 1.** Minimum inhibitory concentration (MIC) and minimum fungicidal concentration (MFC) of liriodenine isolated from *Annona macroprophyllata,* evaluated in 17 fungal isolates.

	Liriodenine			Amphotericin B		Fluconazole	
Fungi	MIC (μg.mL^-1^)	MFC (μg.mL^-1^)	MIC (μg.mL^-1^)	MFC (μg.mL^-1^)	MIC (μg.mL^-1^)	MFC (μg.mL^-1^)
*Paracoccidioides brasiliensis* 339 (ATCC32069)	> 500	> 500	0.5	1	NP	NP
*Paracoccidioides brasiliensis* 18	31.2	31.2	1	1	NP	NP
*Paracoccidioides restrepiensis* 192	62.5	62.5	1	1	NP	NP
*Paracoccidioides lutzii* 01 (ATCC MYA-826)	> 500	> 500	0.5	1	NP	NP
*Paracoccidioides lutzii* 8334	> 500	> 500	1	1	NP	NP
*Paracoccidioides restrepiensis* 234	> 500	> 500	1	1	NP	NP
*Paracoccidioides brasiliensis* 531	250	500	0.5	1	NP	NP
*Paracoccidioides brasiliensis* 326	> 500	> 500	0.5	2	NP	NP
*Histoplasma capsulatum* (ATCC 26029)	1.95	3.90	1	1	NP	NP
*Aspergillus fumigatus* (ATCC 7100)	> 500	> 500	1	1	NP	NP
*Candida albicans* (ATCC 90028)	125	250	1	1	0.25	CC
*Candida glabrata* (ATCC 90030)	125	500	1	1	16	CC
*Candida parapsilosis* (ATCC 22019)	250	500	0.125	0.125	1.0	CC
*Candida krusei* (ATCC 6258)	250	500	1	1	64.0	CC
*Candida tropicalis* (ATCC 750)	> 500	> 500	0.5	0.5	1.0	CC
*Cryptococcus neoformans* (ATCC 28957)	62.5	125	0.25	0.25	8	CC
*Cryptococcus gattii* (ATCC AFLP4)	62.5	125	1	1	4	CC

NP: not performed because it is not the reference antifungal agent; CC: countless colonies.

Among the eight fungal strains of the genus *Paracoccidioides*, only strains Pb18, Pb192 and Pb531 were susceptible to liriodenine, with MICs of 31.2, 62.5 and 250 μg.mL^-1^, respectively. The remaining standard strains and clinical isolates were resistant to the highest concentration evaluated.

Regarding the yeasts used, liriodenine exhibited antifungal activity against most of the strains tested when compared to the amphotericin B and fluconazole controls, except for *Candida tropicalis,* which was found to be resistant to the highest concentration evaluated.

Liriodenine did not exhibit antifungal activity against the *Aspergillus fumigatus* strain. However, the *Histoplasma capsulatum* strain was susceptible to liriodenine at a concentration of 1.95 μg.mL^-1^. The solvent DMSO did not show activity for all strains and clinical isolates evaluated.

### Minimum fungicide concentration

Liriodenine exhibited fungicidal activity against all standard strains and clinical isolates that were sensitive in *in vitro* tests. The MFC was the same as the MIC in all fungi evaluated, with the exception of *Histoplasma capsulatum* and *Candida krusei* (Table 1).

### Scanning electron microscopy

**Figure 3.**
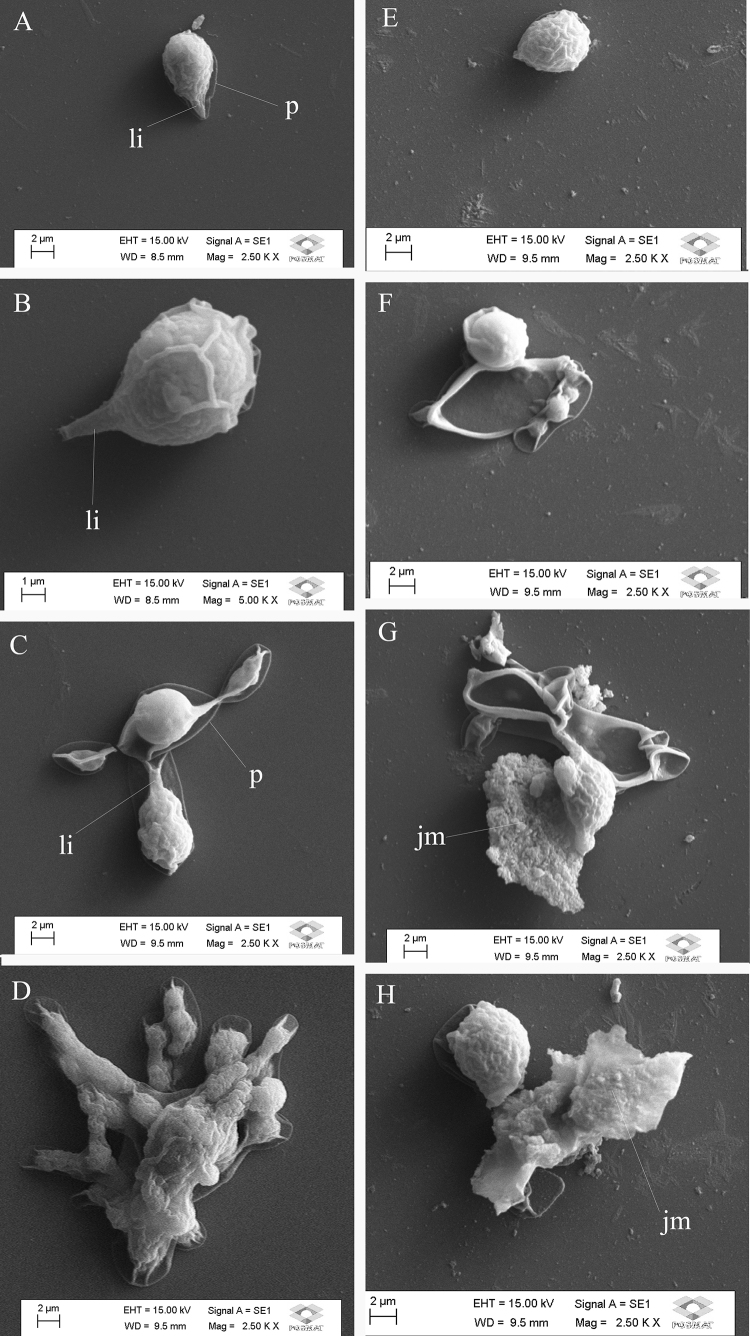
Scanning electron microscopy of yeast-phase cells of standard *Paracoccidioides brasiliensis* (PB18) strain. **(A-D)** Cells not treated with liriodenine. **(E-H)** Cells treated with liriodenine at the MIC of 31.2 μg.mL^-1^. li: ligament; p: prolongation; jm: juxtaposed material.

**Figure 4.**
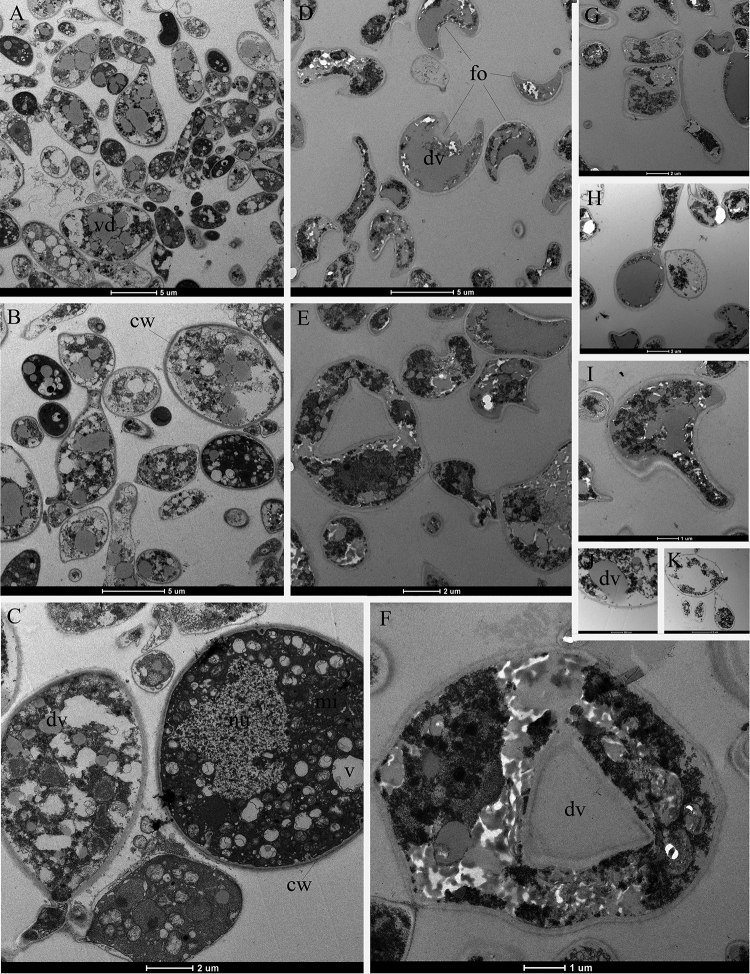
Transmission electron microscopy of yeast-phase cells of the standard *Paracoccidioides brasiliensis* (Pb18) strain. **(A-C)** Cells not treated with liriodenine. **(D-K)** Cells treated with liriodenine at the MIC of 31.2 μg.mL^-1^. dv: dense vacuole; v: electron-lucent; nu: nucleus; mi: mitochondrion; cw: cell wall; fo: sickle shape.

Ultrastructural analysis of yeast-phase cells of *P. brasiliensis* revealed single cells or clusters with several buds (Figs. 3 and 4). SEM of yeast-phase cells not treated with liriodenine showed slightly irregular surfaces and prolongations that attached the cells to the contact surface, which in this case was the culture plate. The prolongations were short, had a regular and delicate morphology, and were visualized close to the cell body, resembling a “halo” around the cell. The single cells exhibited discrete irregularities on their surface that resembled “ribbings”. In addition, a filamentous region (ligament) was detected, which was interpreted as the onset of a bud (Figs. 3A and 3B). Clusters of cells with several ligaments to their buds were noted in other images (Figs. 3C and 3D).

Changes in ultrastructural morphology were observed after treatment with liriodenine at a concentration of 31.2 μg.mL^-1^ (Figs. 3E, 3F, 3G, and 3H). The adhesion “halo” to the culture medium was rarely observed in single cells, although the morphology was similar to that observed in untreated cells in terms of the round shape of the cell and discrete surface irregularities (“ribbings”) (Fig. 3E). However, the most evident ultrastructural alterations were observed in cell clusters, which contained very deformed cells with irregular ligaments, apparently without a lining membrane; plates of material were juxtaposed with these clusters (Figs. 3F, 3G, and 3H).

### Transmission electron microscopy

TEM of cells not treated with liriodenine revealed a round shape, regular contours, and cell walls with uniform thickness. The nucleus and organelles were found to be preserved and intact, and mitochondria, discrete endoplasmic reticula and vacuoles with variable sizes and contents were observed. Some smaller vacuoles were electron-lucent and did not contain visible material; however, most vacuoles observed in the cytoplasm of cells were dense, circular and filled with finely flocculent, homogenous and mildly dense material. Ligaments could be detected between cells and their buds (Figs. 4A, 4B, and 4C).

In contrast, marked changes were found in the morphology of cells treated with liriodenine, as demonstrated by SEM. However, TEM showed details of the cytoplasmic and nuclear alterations. The shape of the cells was altered, and there were only irregular-shaped cells. The cells generally had depressions in their walls, and the plasma membrane exhibited variable shapes, with a sickle shape predominant. Cytoplasmic alterations were very evident and included large cytoplasmic areas without a defined contour filled with finely flocculent, homogenous and mildly dense material, similar to the material observed inside the dense circular vacuoles of the control group. These images suggest the fusion of these various vacuoles in treated cells. In addition, large vacuoles with an irregular, or even triangular, shape containing the same material were noted (Figs. 4D, 4E, 4F, 4G, 4H, 4I, 4J, and 4K).

## Discussion

Nowadays, despite the discovery of new antifungal compounds, some problems persist including low urinary excretion of active drugs, impaired treatment of urinary tract infections, and reduced diffusion into the cerebrospinal fluid regardless of the degree of meningeal inflammation. Moreover, interactions with different drugs and restrictions concerning treatment of pregnant women also constitute limitations [40-43]. These difficulties also justify continuous research to identify new antifungal compounds, including those of plant origin.

The use of plant extracts for the treatment and prophylaxis of diseases is one of the oldest practices of humans. The search for new plant-derived drugs has developed substantially in recent years due to increasing resistance to antimicrobial compounds, undesirable effects of existing compounds and drug interactions [44,45]. The main advantage of this approach over modifications in already existing compounds is the chance of identifying new prototypes of drugs with clearly different chemical structures, thus avoiding cross-resistance and currently observed drug interactions [31].

Few studies have evaluated the activity of plant-derived compounds against pathogenic fungi. The present study demonstrated the antifungal activity of liriodenine against three fungal strains of the genus *Paracoccidioides*; however, greater activity was observed against yeasts of the genus *Cryptococcus* and against the thermally dimorphic fungus *Histoplasma capsulatum*. Among the studies focusing on the genus *Paracoccidioides*, two extracts derived from the genus *Annona* [46,47] and three evaluated compounds extracted from other plant species [48-50] were used. In this respect, Lima et al. [46] evaluated the activity of fatty acid methyl esters (FAMEs) against 12 clinical isolates of *P. brasiliensis* by the broth microdilution method and observed inhibition of all isolates, with MIC values ranging from 3.4 to 55.5 μg.mL^-1^. This MIC range was similar to that observed in the present study. In another study, Lima et al. [47] investigated the activity of acetogenins extracted from *Annona cornifolia* against Pb01 and Pb18 by the broth microdilution method. Concentrations of 1.17 to 150 μg.mL^-1^ were tested. Acetogenins exhibited activity against the two strains at concentrations ≤ 150 μg.mL^-1^ and were found to be more active than the combination of sulfamethoxazole and trimethoprim, but less active than amphotericin B.

Using the dry weight method, San-Blas et al. [48] demonstrated that ajoene, a sulfur compound found in garlic (*Allium sativum*), inhibited the growth of *P. brasiliensis* by 90% in the yeast phase and by 60% in the mycelial phase at a concentration of 11.7 μg.mL^-1^. Electron microscopy revealed that 11.7 μg.mL^-1^ of ajoene affected the integrity of the fungal cytoplasmic membrane, but cell lysis was only observed at a concentration of 46.8 μg.mL^-1^.

The hexane fractions of hydroalcoholic extracts of *Baccharis dracunculifolia* and *Piper regnellii* exhibited antifungal activity against three strains of the genus *Paracoccidioides* (Pb18, Pb01 and Pb339). Compounds from *Baccharis dracunculifolia* DC were active against Pb18, Pb01 and Pb339, with MICs of 7.8, 7.8 and 30 μg.mL^-1^, respectively, while the MIC of compounds from *Piper regnellii* was 7.8 μg.mL^-1^ for the three strains evaluated [49]. Comparison of these results with those of the present study shows that strain Pb339 is resistant to liriodenine but susceptible to the hexane fractions, while strain Pb18 is more sensitive to liriodenine. Furthermore, the compounds maytenin and pristimerin derived from *Maytenus ilicifolia* exhibited antifungal activity against yeasts and filamentous fungi. Strain Pb18 was sensitive to both compounds at a concentration of < 0.12 mg/L. Maytenin provided better results than pristimerin. No cytotoxic effects of these compounds on oral mucosa keratinocytes were observed, suggesting an adequate safety level [50].

The ultrastructural data of yeast-phase cells of *P. brasiliensis* treated with liriodenine revealed alterations mainly in the cell wall and, to a lesser extent, alterations in the cytoplasm. Similar findings have been reported for ajoene, including cell wall damage, cell lysis and deterioration of the structures in yeast-phase cells of *P. brasiliensis* [48].

This study revealed a MIC of liriodenine against *Histoplasma capsulatum* ATTC 26029 of 1.95 μg.mL^-1^. Alanís-Garza et al. [51] demonstrated the antifungal activity of hexane, ethyl acetate and butanol extracts isolated from 15 plants in the Northeastern region of Mexico against *Histoplasma capsulatum* had MICs ranging from 16 to 1.000 μg.mL^-1^. Even lower MIC values were observed for maytenin (0.97 mg/L) and pristimerin (0.48 mg/L) [50].

In recent years, an increase in invasive infections caused by yeasts of the genus *Candida* has been observed, especially among immunosuppressed patients [52]. *Candida albicans* is the main causative agent of candidemia, although the prevalence of non-*Candida albicans* etiology has been increasing [53]. The susceptibility of *Candida* species to commonly used antifungal agents is variable; *C. krusei* exhibits intrinsic resistance to fluconazole [54], while *C. glabrata* shows acquired resistance [55].

In the present study, liriodenine exhibited antifungal activity against most *Candida* strains, with MICs of 125 to 250 μg.mL^-1^, except for *C. tropicalis,* which was resistant to the highest concentration evaluated. These values are similar to those reported for pristimerin [50], with MICs ranging from 7.81 to 250 mg/L, and for the hexane extract of *Annona glabra* [56], which inhibited the growth of *C. albicans* at a concentration of 100 μg.mL^-1^. The present study also showed that liriodenine has lower MIC values than the methanol and ethyl acetate extracts of *Duguetia furfuracea* against *C. albicans* and *C. krusei*, with MICs ≥ 1024 μg.mL^-1^, although acting against *C. tropicalis* [57]. Liriodenine was also more active than FAMEs of *Annona cornifolia*, which exhibited MICs of 500 μg.mL^-1^ or higher against *C. albicans* and *C*. *parapsilosis* strains [46]. Finally, the MIC values presented by liriodenine against *Candida* species ranged between 125 and 250 μg.mL^-1^ while these values were much lower, 3.12 μg.mL^1^, for the same substance, but extracted from *Liriodendron tulipifera*, *Magnoliaceae* [31]. This difference can be explained by the plant liriodenine was extracted as well as phase of its development. 

Cryptococcosis is one of the main mycoses that affects immunosuppressed patients, especially those infected with human immunodeficiency virus, because of the severity of clinical manifestations, the incidence and intensity of sequelae, and the high mortality rate [58]. Cryptococcal meningitis should be treated with a combination of amphotericin B or its lipid derivatives with flucytosine. However, classical amphotericin B requires intravenous administration and careful control of side effects. The lipid derivatives of amphotericin B are less toxic but are expensive and require intravenous administration. Finally, flucytosine is not available in many countries, including Brazil. These difficulties have led to the use of high doses of fluconazole, which is fungistatic and provides slow clearance of infection. This scenario encouraged the investigation of other therapeutic regimens, including available antifungal compounds, the combination of antifungals with immunomodulators, and the search for other drugs with anticryptococcal activity [59-62].

In the present study, liriodenine exhibited activity against isolates of two complexes, *Cryptococcus neoformans* complex and *C. gattii* complex, with an MIC of 62.5 μg.mL^-1^ for both species. The MIC observed in this study was much lower than the MICs for FAMEs of *Annona cornifolia* [54] (≥ 500 μg.mL^-1^) and lower than three compounds from *Ocimum basilicum* - essential oil, crude ethanol extract and hexane fraction (MICs of 156 to 2.500 μg.mL^-1^) [63]. However, the MICs of maytenin (0.48 to 3.9 mg/L) and pristimerin (0.97 to 7.8 mg/L) were even lower [50].

The MICs of amphotericin B and fluconazole on *Candida* species and *Cryptococcus* species, used as controls, were very similar to those that had been published by other authors [64-67].

*Aspergillus fumigatus* is the main etiological agent of invasive aspergillosis that affects neutropenic patients [68,69]. Treatment is performed mainly with triazole compounds such as voriconazole and posaconazole [70]. However, surveillance studies have revealed the emergence of resistant clinical and environmental isolates worldwide, [71-76] particularly in patients previously using azole compounds or infected with fungi exposed to azole derivatives used in agriculture [77,78]. In the present study, liriodenine did not exert antifungal activity against the *Aspergillus fumigatus* strain. On the other hand, maytenin at a concentration of 125 mg.L and pristimerin at a concentration of 250 mg/L were found to be active against this fungus, with maytenin exhibiting the best fungicidal activity [50].

The mechanism of the antifungal action of liriodenine has not yet been clarified. *In vitro* and *in vivo* studies demonstrated that liriodenine inhibits topoisomerase II, an enzyme that induces DNA double strand breaks and is necessary to relieve torsional stress during replication. The antifungal action of liriodenine maybe similar to that of antibiotics that inhibit this enzyme, leading to defective synthesis of messenger RNA and proteins and consequent death of the microorganism [79].

Liriodenine inhibits the biosynthesis of dopamine and dopamine content induced by L-DOPA in PC12 cells [80]. Since melanin, an important virulence factor of *Cryptococcus* species, is also synthesized from L-DOPA, it would be interesting to investigate the possible antimelanogenic activity of liriodenine.

However, a study using *Saccharomyces cerevisiae* suggested another mechanism of action of liriodenine on yeasts. That study showed that liriodenine interrupts mitochondrial iron-sulfur synthesis based on three findings:

- liriodenine caused cellular iron imbalance through the induction of genes that are important for iron uptake and cellular iron homeostasis;
- genome analysis demonstrated that yeast mutants with deletions in the genes responsible for iron homeostasis are hypersensitive to liriodenine;
- treatment of yeasts with liriodenine produced cellular defects that resembled deficiencies in mitochondrial iron-sulfur synthesis, including an increase in mitochondrial iron levels, a decrease in the activity of sulfur enzymes, and an increase in oxidative stress [81].

Biochemical models designed to determine the mechanism of action of antifungal compounds should be used for the evaluation of liriodenine [82,83].

## Conclusion

The present *in vitro* study demonstrated the fungicidal activity of liriodenine against yeasts and some dimorphic fungi. Therefore, it is believed additional evaluation of this compound in animal models of infection should be carried out, followed by the estimation of its safety and efficacy for the treatment of human infections. Our initial findings also encouraged the modification of liriodenine chemical structure, in order to turn it into a more effective product.
